# Inflammatory Neovascularization and Vascular Remodeling Associated With Carotid Plaque Destabilization

**DOI:** 10.1002/cns.70992

**Published:** 2026-06-19

**Authors:** Xuejiao Dai, Jia Huang, Feihua Ni, Ruying Hu, Huiyan Zheng, Dan Wang, Jianqiao Zhang, Ting Sun

**Affiliations:** ^1^ Department of Health Management Center, Second Affiliated Hospital, School of Medicine Zhejiang University Hangzhou Zhejiang China

**Keywords:** carotid plaques, endothelial cells, neovascularization, vascular remodeling, vascular smooth muscle cells

## Abstract

**Aims:**

Inflammatory responses promote pathological neovascularization and vascular remodeling in carotid plaques, contributing to plaque destabilization. This study aims to clarify specific cellular subpopulations that mediate pathological neovascularization and vascular remodeling in different plaque regions.

**Methods:**

This study systematically analyzed the cellular composition and functional distribution in the atherosclerotic core (AC) and proximal adjacent (PA) regions of carotid plaques using single‐cell RNA sequencing.

**Results:**

We observed that a subset of endothelial cells contributing to pathological neovascularization was prominent in the AC region, while the PA region exhibited a reduction in contractile vascular smooth muscle cells (VSMCs) and an increase in osteogenic VSMCs, collectively contributing to vascular remodeling. Additionally, fibroblast‐mediated inflammatory regulation was predominantly enriched in the PA region, whereas the AC region harbored more macrophage subsets involved in lipid metabolism and immune responses.

**Conclusion:**

The pathological neovascularization mediated by endothelial cell subpopulations in the AC region, along with the reduced contractile VSMCs and increased osteogenic VSMCs in the PA region, is a factor that promotes plaque instability.

## Graphical abstract

A subset of endothelial cells in the AC region promoted neovascularization, while the PA region exhibited an increase in osteogenic VSMCs, contributing to vascular remodeling. Fibroblast‐mediated inflammatory regulation was predominantly enriched in the PA region, whereas the AC region harbored more macrophage subsets involved in lipid metabolism and immune responses.

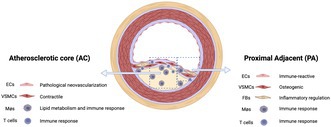

## 1 Introduction

Rupture of atherosclerotic plaques represents a major trigger for acute cardiovascular and cerebrovascular events, accounting for over 20.5 million deaths annually worldwide [1, 2, 3]. Lipid accumulation within atherosclerotic plaques initiates inflammatory responses that lead to endothelial cells (ECs) injury and vascular smooth muscle cells (VSMCs) dysfunction, collectively compromising vascular integrity. Subsequent pathological neovascularization and phenotypic switching of smooth muscle cells contribute to vascular remodeling. However, these newly formed vessels exhibit marked fragility and hemorrhagic propensity, paradoxically exacerbating plaque instability and rupture risk, thereby increasing the likelihood of cerebral infarction [4, 5].

Previous studies have demonstrated that intraplaque neovascularization primarily originates from the vasa vasorum in the adventitia as a hypoxic response to increased oxygen demand in developing plaques [6, 7]. These neovessels are prone to rupture due to structural defects, leading to intraplaque hemorrhage [8]. In addition, insufficient pericyte coverage increases vascular permeability, promoting lipoprotein extravasation and cholesterol deposition. Immature plaque vessels may be the main source of new inflammatory foci [9, 10], serving as channels for inflammatory cells (such as macrophages and lymphocytes) that release matrix metalloproteinases (MMPs). These enzymes degrade the collagen fibers supporting the plaque, ultimately thinning the fibrous cap [8]. Furthermore, VSMCs undergo phenotypic switching under chronic inflammatory stimulation induced by plaque lipids, actively participating in vascular remodeling processes. VSMCs localized in the fibrous cap region predominantly exhibit a synthetic or dedifferentiated phenotype, demonstrating enhanced proliferative capacity, migratory potential, and extracellular matrix (ECM) secretory activity that collectively contribute to plaque restructuring. These cells concomitantly release chemokines and cytokines, thereby establishing a pro‐inflammatory microenvironment that further activates adjacent cellular components [11, 12, 13]. The osteoblast‐like and chondrocyte‐like VSMCs are primarily distributed within the fibrous cap of atherosclerotic plaques or in chondroid metaplasia and calcified regions, playing a key role in plaque calcification [14]. These heterogeneous VSMC phenotypes collectively contribute to the vascular remodeling process.

Several drugs targeting neovascularization and vascular remodeling have been developed. Targeted inhibition of VEGF signaling can reduce vascular permeability in early‐stage plaques (IPN grades I–II) by suppressing EC proliferation. However, this approach demonstrates limited efficacy against mature plaques (IPN grades III–IV), and systemic treatment may interfere with normal vascular repair functions [15, 16]. Therefore, the identification of specific neovascular endothelial or VSMC subpopulations and the development of targeted regulatory strategies against these subpopulations constitute a crucial research focus and potential therapeutic breakthrough.

Single‐cell sequencing provides a new perspective for the analysis of subgroups of cells in carotid plaques. A single‐cell sequencing study (GSE159677) of the atherosclerotic core (AC) and proximal adjacent (PA) regions of human carotid plaques focuses on the key factors that drive chronic inflammation in ECs and VSMCs [17]. The primary objective of this study is to identify distinct EC subpopulations associated with intraplaque neovascularization, characterize VSMC phenotypic heterogeneity, and delineate compartmentalized immune response signatures in both AC and PA regions through single‐cell RNA sequencing analysis of patient‐derived carotid plaques, thereby providing potential therapeutic targets for subsequent investigation.

## 2 Results

### 2.1 Single‐Cell Profiling of Vascular and Immune Cell Subpopulations in AC and PA Regions of Carotid Artery Plaques

**FIGURE 1:**
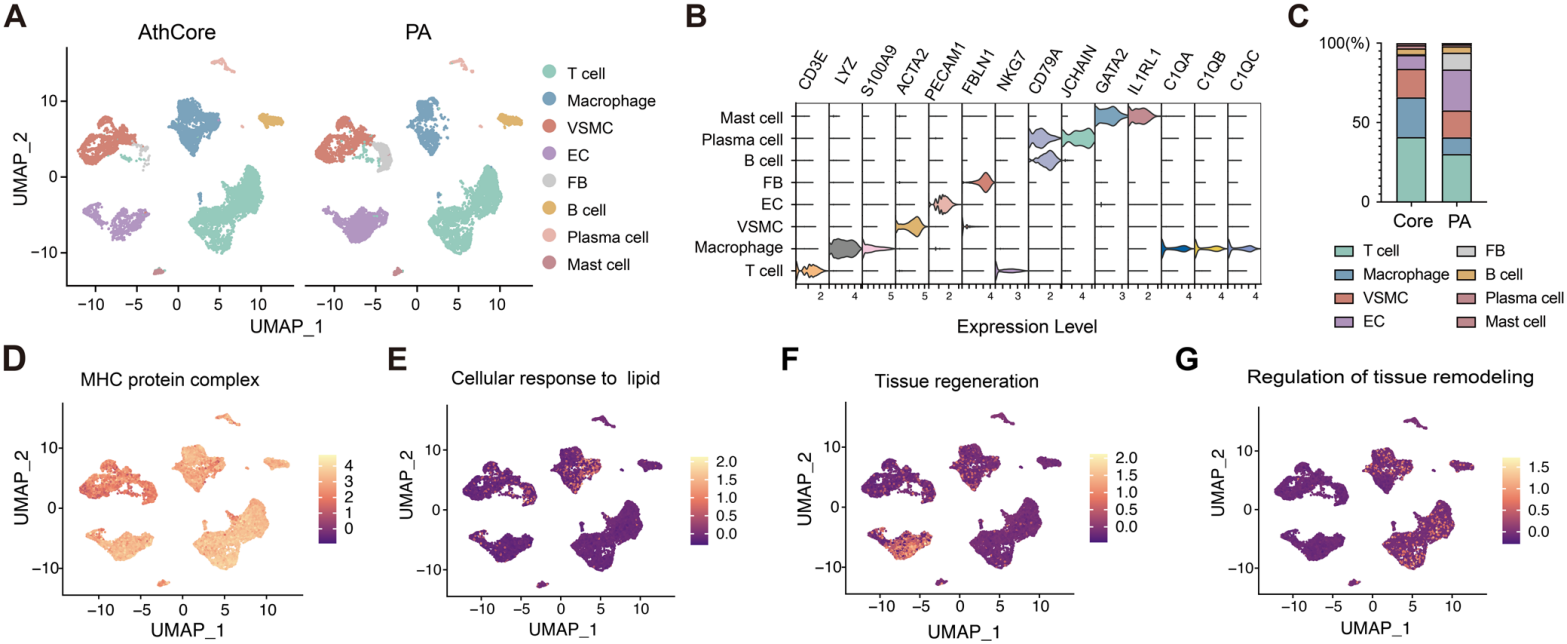
Heterogeneous distribution and functional differences of cell subpopulations in carotid PA tissue and AC regions. (A) UMAP plot of eight major cell types in AC and PA plaques. (B) Violin plot of marker gene expression for the eight cell clusters. (C) Bar plots showing the proportions of the different cell subsets. (D–G) Scaled module score based on functional gene sets.

The necrotic core and proximal adjacent regions of carotid atherosclerotic plaques exhibit distinct pathological neovascularization and vascular remodeling. To investigate the molecular and cellular alterations of vascular and immune cell subpopulations in AC and PA regions, we explored the transcriptomic dataset (GSE159677) of single cells derived from human atherosclerotic plaques and their patient‐matched PA carotid segments. We identified eight distinct cell populations, including T cells, macrophages, VSMCs, ECs, fibroblasts (FBs), B cells, plasma cells, and mast cells (Figure 1A,B). The proportions of T cells (40.89% vs. 30.27%) and macrophages (25.03% vs. 10.48%) were higher in the core region compared to proximal adjacent tissue, whereas ECs (8.69% vs. 25.77%) and FBs (0.45% vs. 10.95%) were less abundant in the core region (Figure 1C).

To investigate the contribution of these cell populations in vascular remodeling, we calculated module scores for each group based on functional gene sets (Figure 1D–G, Figure S1A–C). All cell types exhibited high scores for MHC protein complex‐related functions, indicating activated antigen presentation (Figure 1D). Particularly, three major cell types including ECs, macrophages, and T cells showed higher gene expression involving leukocyte chemotaxis (Figure S1A). And macrophages exhibited upregulated lipid metabolism functions (Figure 1E). In addition, vascular ECs showed enhanced tissue regeneration functions (Figure 1F), while VSMCs displayed upregulated ECM assembly functions (Figure S1B) and sprouting angiogenesis (Figure S1C). Immune cells, including macrophages, T cells, and B cells, were involved in regulating tissue remodeling (Figure 1G). These findings reveal that vascular ECs and VSMCs might serve as primary mediators of vascular remodeling, whereas immune cells exhibit a dual function in both driving inflammation and promoting tissue repair.

### 2.2 A Pro‐Pathological Angiogenic Type of ECs Exists in the AC Region, and Immune‐Reactive ECs Are Significantly Upregulated in the PA Tissue

**FIGURE 2:**
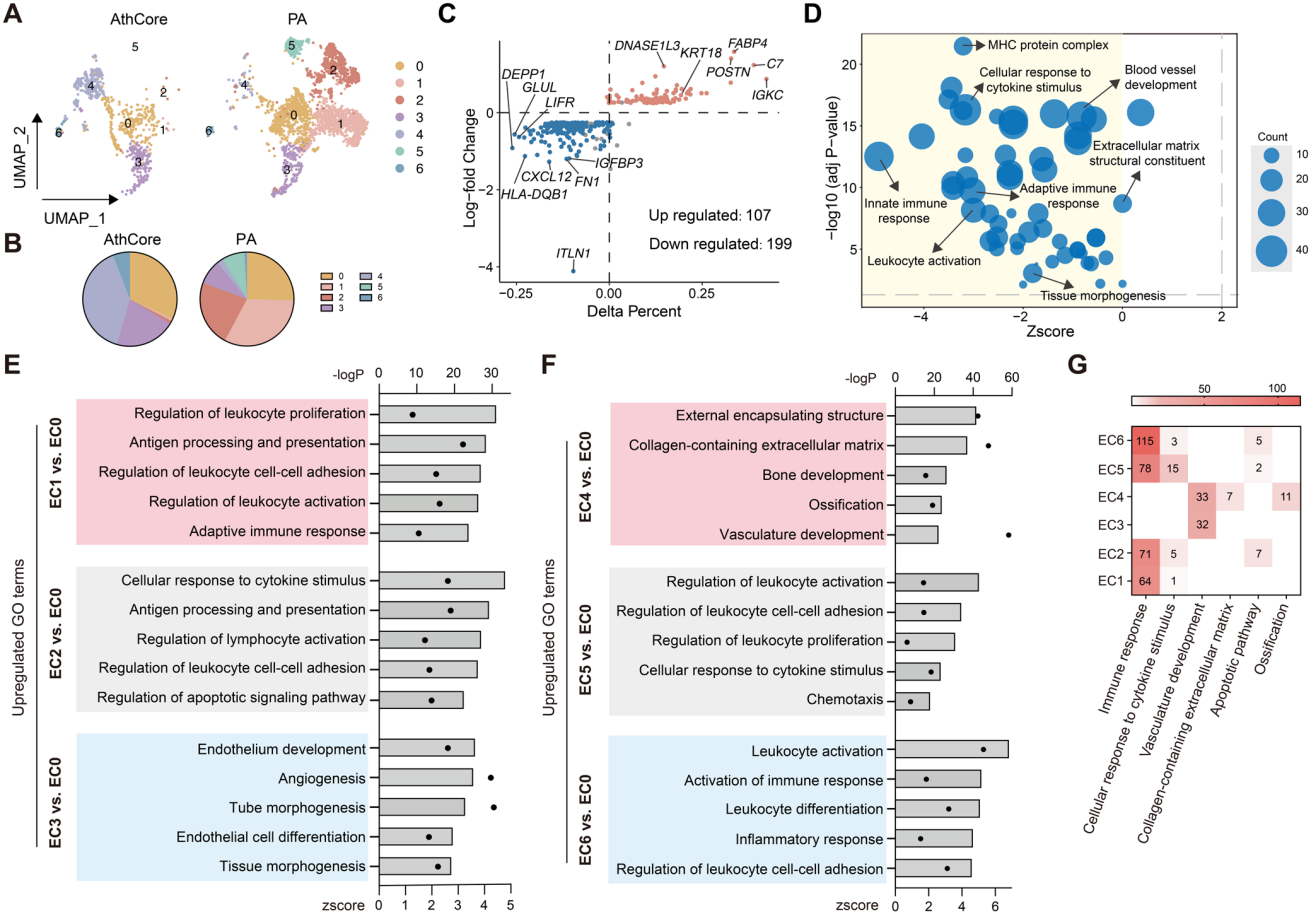
Immune‐reactive endothelial cells are significantly upregulated in PA tissue. (A) UMAP plot of ECs in the AC and PA regions. (B) Proportions of the different ECs subsets. (C) Scatter plot showing the upregulated genes (pink) and downregulated genes (blue) in the EC0 compared to other EC subsets. (D) Bubble plot of GO *z*‐score and −log10 (adj *p*‐value) in the EC0 compared to other EC subsets. (E, F) Bar plot displaying upregulated GO terms in different EC subsets compared to EC0. Bars represent z‐score values, and dots represent −log10(adjusted *p*‐value). (G) Number of upregulated GO terms in different EC subsets compared to EC0.

Vascular ECs are the crucial inner lining of blood vessels, playing a pivotal role in vascular homeostasis and integrity [18]. UMAP visualization of ECs revealed distinct distributions between the AC and PA regions (Figure 2A and Figure S2A). The proportion of EC4 was significantly higher in AC region, whereas EC1, EC2, and EC5 were more abundant in PA region (Figure 2B). Comparing EC0 to the other EC subpopulations, we identified 107 upregulated genes (e.g., *FABP4*, *C7*, *POSTN*, *IGKC*, *DNASE1L3*, *KRT18*), involving in enhanced lipid metabolism (*FABP4*), angiogenesis (*POSTN*), and complement function (*C7*). Conversely, 199 genes were downregulated (e.g., *ITLN1*, *HLA‐DQB1*, *CXCL12*, *FN1*, *IGFBP3*), indicating reduced immune response (*HLA‐DQB1*), vasculature development (*CXCL12*), inflammatory regulation (*ITLN1*), metabolic/oxidative stress response (*IGFBP3*), and ECM remodeling (*IGFBP3*, *FN1*, *ITLN1*) (Figure 2C). GO analysis suggested that EC0 exhibited overall downregulation in leukocyte activation, immune response, tissue angiogenesis, and ECM remodeling, suggesting it may represent a basal state (Figure 2D). Genes typically low in quiescent ECs (*ASS1*, *IFNGR1*, *OPTN*) were also minimally expressed in EC0 (Figure S2B). These features indicate that the EC0 subcluster may be in a resting state, which is consistent with the findings of other previous studies [19].

Comparing other EC subpopulations to EC0, we found that EC1 was enriched in leukocyte proliferation, activation, adhesion, and antigen presentation (Figure 2E). EC2 showed additional upregulation in cytokine response and apoptotic metabolic pathways (Figure 2E). EC3 exhibited enhanced endothelial development, differentiation, and angiogenesis (Figure 2E). EC4 also displayed angiogenic functions but was more prominently associated with ECM remodeling and calcification (Figure 2F), suggesting pathological angiogenesis [20]. EC5 showed elevated responses to cytokines and chemokines alongside leukocyte regulation (Figure 2F). EC6 was enriched in leukocyte differentiation, immune‐inflammatory activation, and adhesion (Figure 2F). Based on their functional features, we found that EC1, 2, 5, and 6 were involved in immune response, with EC2 showing 7 apoptosis‐related terms, EC5 exhibiting 15 cytokine response terms, and EC6 displaying 115 immune response terms (Figure 2G). EC1, 2, and 5 were predominantly localized in the PA (Figure 2A), suggesting heightened immune activity in the PA tissue. Both EC3 and EC4 were associated with angiogenesis, and EC4 uniquely contributed to ECM remodeling and ossification (Figure 2G). EC4 was primarily found in the AC region (Figure 2A), indicating pathological angiogenesis in the plaque core.

**FIGURE 3:**
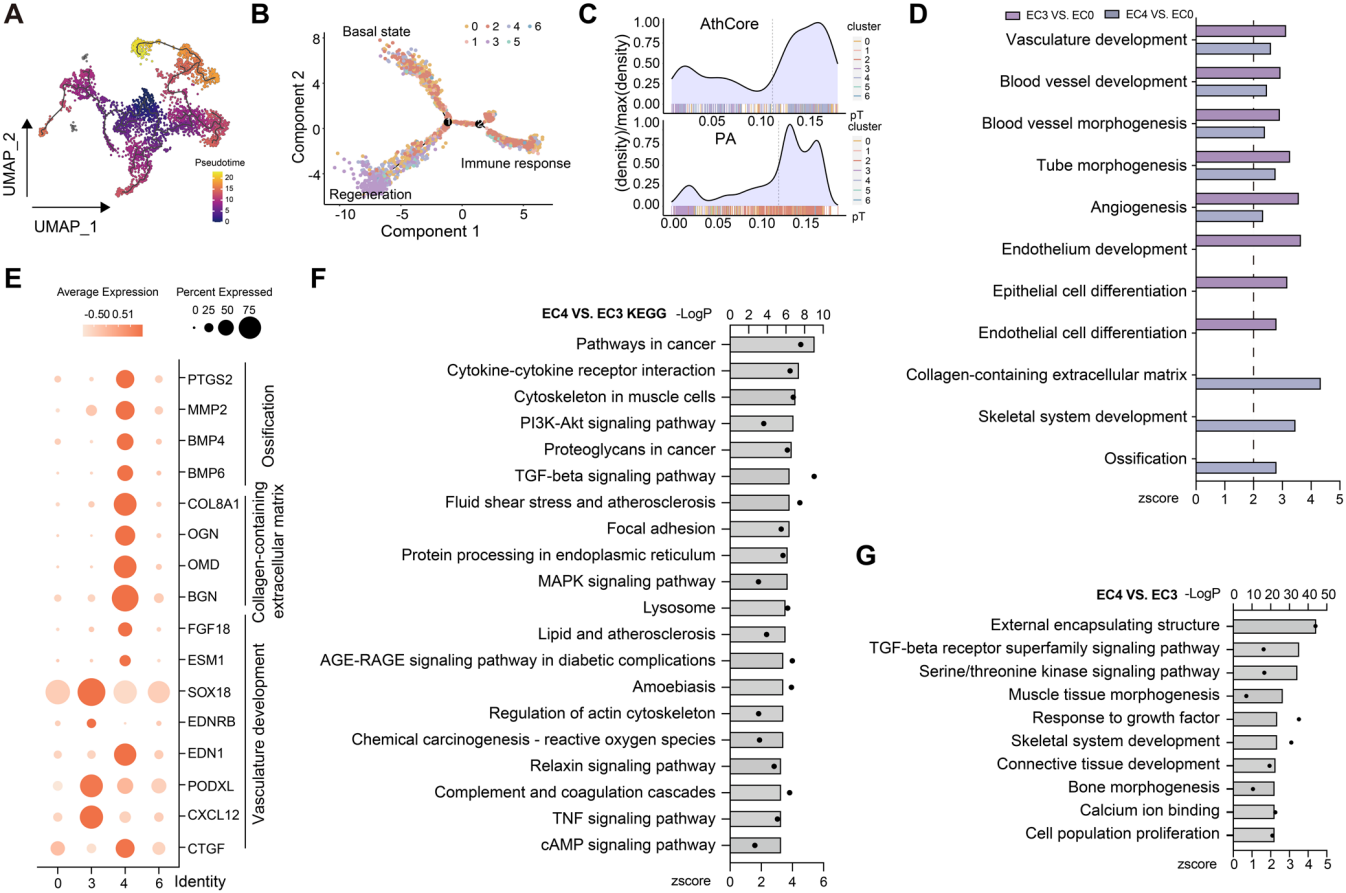
A pro‐pathological angiogenic endothelial subpopulation exists in the AC region. (A) Differentiation trajectory of EC subsets. (B) Inferred differentiation trajectory of ECs with monocle2. (C) The distribution of ECs on pseudotemporal axes in the indicated groups. Color‐coded bars indicate the positions of ECs on pseudotemporal axes. (D) Bar plot displaying upregulated GO terms in EC3 and EC4 subsets compared to EC0. Bars represent *z*‐score values, and dots represent −log10(adjusted *p*‐value). (E) Dot plot displaying marker genes related to angiogenesis in the indicated groups. (F) Upregulated KEGG pathway in EC4 compared to EC3. Bars represent *z*‐score values, and dots represent −log10(adjusted *p*‐value). (G) Bar plot displaying upregulated GO terms in EC4 compared to EC3. Bars represent *z*‐score values, and dots represent −log10(adjusted *p*‐value).

To further explore the function of the EC subsets, we performed pseudotime analysis which revealed three developmental trajectories for ECs, including EC0 → EC1 → EC2 → EC5 (inflammation), EC0 → EC4 → EC6 (neovascularization), and EC0 → EC3 (angiogenesis) (Figure 3A). Three endpoints were defined by monocle2 trajectory analysis, including a basal state, an immune‐response state, and a regenerative state (Figure 3B). ECs in the AC region trended toward regeneration, whereas those in the PA region leaned toward immune response (Figure 3C). For the inflammation direction, heatmap analysis of immune‐inflammatory subpopulations showed differential gene expression across pseudotime stages (Figure S3A). ECs in the PA region expressed late‐stage genes more prominently (Figure S3B). EC5 exhibited the highest expression of the chemokine *CXCL2*, while EC1, EC2, and EC5 showed elevated antigen presentation genes (*HLA‐DQB1*, *HLA‐DQA1*, *CD74*) (Figure S3C–E). To compare the differences in the roles of EC3 and EC4 in vascular remodeling, we observed the functions of these two cell subpopulations. Unlike the angiogenic role of EC3, EC4 primarily functions in ECM, skeletal system development, and ossification (Figure 3D). Vasculature development genes (*SOX18*, *EDNRB*, *PODXL*, *CXCL12*) [21, 22, 23, 24] were highly expressed in EC3, whereas EC4 additionally expressed ECM‐related (*COL8A1*, *OGN*, *OMD*, *BGN*) and calcification‐related (*PTGS2*, *MMP2*, *BMP4*, *BMP6*) genes (Figure 3E). Immunofluorescence staining also revealed that COL8A1 was highly expressed in CD31^+^ ECs within the AC region, but showed low expression levels in ECs of the PA tissues. CXCL12 was highly expressed in CD31^+^ ECs in the plaque core, and also exhibited partial expression in ECs of the PA tissues (Figure S3F). KEGG pathway analysis indicated that EC4, compared to EC3, upregulated pathways such as cancer, cytokine‐cytokine receptor interaction, muscle cytoskeleton, PI3K‐Akt signaling, proteoglycans in cancer, and TGF‐β signaling (Figure 3F). GO analysis highlighted EC4 enrichment in external encapsulating structure, TGF‐β pathway, serine/threonine kinase activity, muscle/bone morphogenesis and calcium ion binding (Figure 3G). These findings suggest that EC4 drives pathological angiogenesis in the plaque core, exacerbating plaque instability.

### 2.3 Contractile‐Phenotype VSMCs Are Predominantly Localized in the AC Region, While Osteogenic Phenotype VSMCs Are Enriched in the PA Region

**FIGURE 4:**
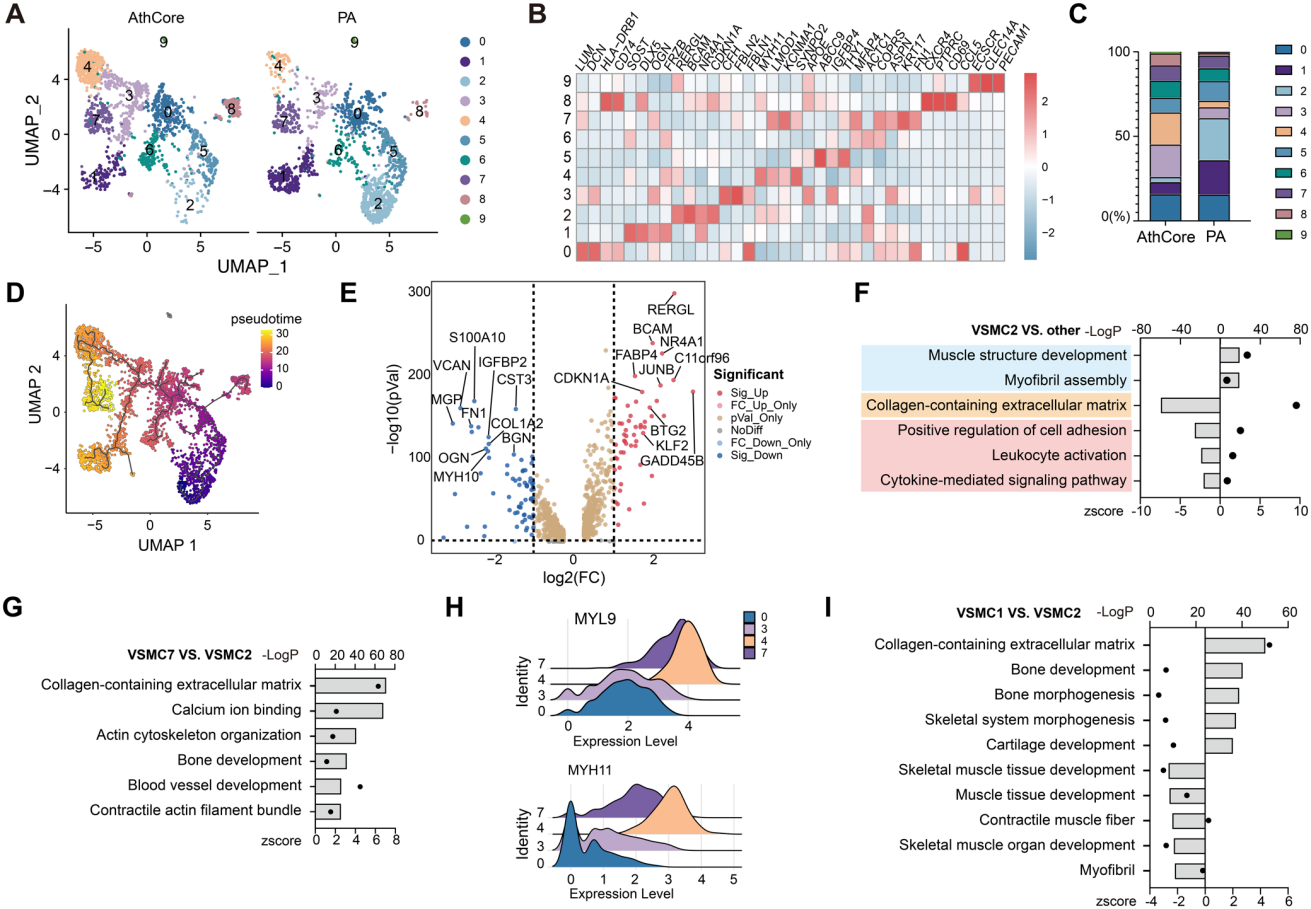
Contractile‐phenotype VSMCs are predominantly localized in the AC region, while osteogenic‐phenotype VSMCs are enriched in the PA region. (A) UMAP plot of VSMCs in the AC and PA regions. (B) Heatmap showing the marker gene expression for each VSMCs subpopulation. (C) Proportions of the different VSMCs subsets. (D) Differentiation trajectory of VSMCs subsets. (E) Scatter plot showing the upregulated genes (red) and downregulated genes (blue) in the VSMC2 compared to other VSMC subsets. (F) Bar plot displaying upregulated and downregulated GO terms in the VSMC2 compared to other VSMC subsets. Bars represent *z*‐score values, and dots represent −log10(adjusted *p*‐value). (G) Bar plot displaying upregulated GO terms in the VSMC7 compared to VSMC2 subsets. Bars represent z‐score values, and dots represent −log10(adjusted *p*‐value). (H) Ridge plot of different gene expression related to contraction in the indicated VSMC subsets. (I) Bar plot displaying upregulated and downregulated GO terms in the VSMC1 compared to VSMC2 subsets. Bars represent *z*‐score values, and dots represent −log10(adjusted *p*‐value).

VSMCs in normal tunica media express a series of contractile markers, such as α‐actinin‐2 (*ACTA2*/*α‐SMA*), smooth muscle cell myosin heavy chain 11 (*MYH11*/*SMMHC*) [12]. However, in atherosclerosis lesions, VSMCs undergo phenotypic transformation into mesenchymal‐like cells, foam cells, macrophage‐like cells, and osteogenic cells [25, 26, 27, 28, 29, 30]. Furthermore, the migration and proliferation capacity of VSMCs were enhanced, and more ECM proteins and cytokines were secreted [31]. Based on enriched genes, VSMCs were classified into 10 subpopulations (Figure 4A,B). UMAP visualization revealed distinct distributions of VSMC subpopulations between the AC and PA regions (Figure 4A). VSMC4 and VSMC8 were significantly more abundant in the AC region, whereas VSMC1 and VSMC2 were predominantly found in the PA region (Figure 4A,C).

Pseudotime analysis indicated that VSMCs originated from VSMC2 and there are mainly four endpoints including VSMC8, VSMC6, VSMC1, and VSMC3‐VSMC4‐VSMC7 (Figure 4D). To investigate the function of these VSMCs, we performed functional enrichment analysis. Compared to the other subpopulations, VSMC2 exhibited upregulated expression of *RERGL* (function unclear), *BCAM* (adhesion), *NR4A1* (anti‐inflammatory, anti‐proliferative, pro‐contractile), *FABP4* (lipid metabolism), *JUNB* (anti‐proliferative, pro‐contractile), *BTG2* (anti‐proliferative, pro‐contractile), *KLF2* (contractile maintenance), *CDKN1A*, and *GADD45B*, while downregulating *MYH10* (contraction), ECM‐related genes (*OGN*, *BGN*, *COL1A2*, *FN1*, *MGP*, *VCAN*), *IGFBP2* (proliferation), and *S100A10* (migration) (Figure 4E). GO analysis suggested that VSMC2 displayed upregulated functions in muscle structure development and myofibril assembly but downregulated ECM organization, cell adhesion, and leukocyte activation (Figure 4F), indicating that VSMC2 may represent a basal VSMC state [32].

Compared to the basal VSMC2, VSMC7 showed significant upregulation in ECM remodeling, calcium ion binding, and bone morphogenesis, suggesting its role in calcified plaque formation (Figure 4G). Additionally, VSMC7 exhibited moderate upregulation in actin cytoskeleton organization and contractile actin filament bundle (Figure 4G). Pseudotime analysis positioned VSMC4 as a precursor to VSMC7, with VSMC4 being highly abundant in the AC region (Figure 4A,C). Analysis of contractile genes (*MYH11*, *MYL9*) in this trajectory confirmed that VSMC4 represents a classic contractile phenotype (Figure 4H), supported by GO terms such as contractile muscle fiber and muscle cell development (Figure S4A). In contrast, VSMC7 demonstrated enhanced synthetic functions (Figure 4G) and slightly increased contractility (Figure 4H), suggesting a potential mesenchymal transition. Keratin 7 (*KRT7*), a gene promoting mesenchymal transition in other diseases [33, 34], was highly expressed in VSMC7, further supporting this shift (Figure S4B). As another endpoint, the VSMC1 group is mainly distributed in the PA area (Figure 4A). VSMC1, compared to the basal VSMC2, showed marked upregulation in ECM and bone development but downregulation in skeletal muscle tissue development and contractile muscle fiber, implicating its osteogenic phenotype and role in plaque calcification (Figure 4I). Vascular calcification is a tightly regulated process, primarily driven by VSMCs developing an osteochondrogenic phenotype, and is associated with increased CVD risk [35]. Notably, both inhibitory (*FRZB*, *SOST*) and promotive (*DLX5*, *DLX‐AS1*) calcification genes were highly expressed in VSMC1, reflecting a complex regulatory network (Figure S4C).

In summary, the contractile VSMCs are mainly distributed in the AC area, while in the PA area, the contractile VSMCs decrease and the osteogenic VSMCs increase. This suggests that VSMCs in the PA area play an important role in vascular remodeling in atherosclerotic plaques.

### 2.4 Fibroblasts Are Predominantly Localized in the PA Region, With Subsets Exhibiting Inflammation‐Related Phenotypes

**FIGURE 5:**
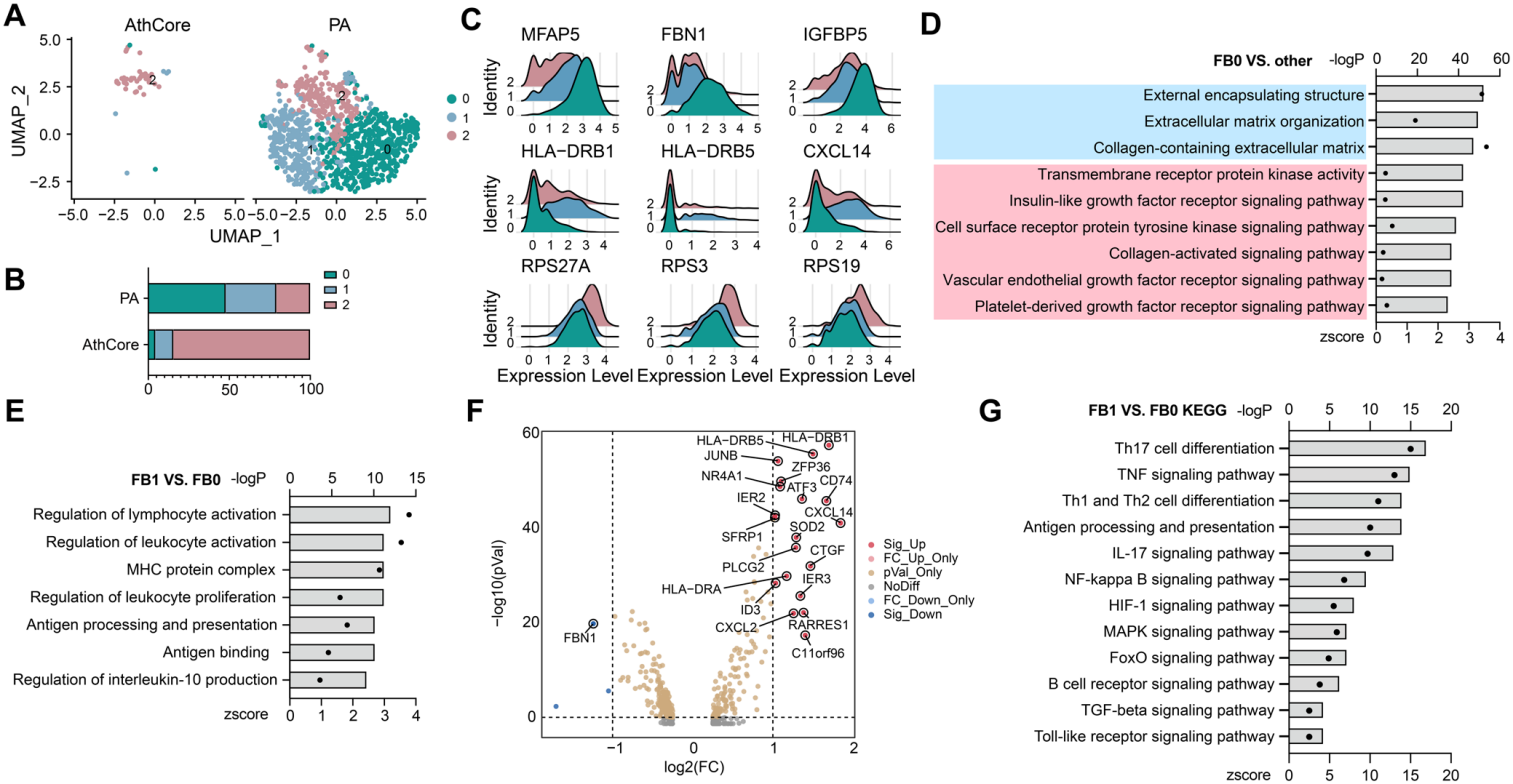
Fibroblasts are predominantly localized in the PA tissues, with subsets exhibiting inflammation‐related phenotypes. (A) UMAP plot of FBs in the AC and PA regions. (B) Proportions of the different FBs subsets. (C) Ridge plot of different gene expression in the indicated FBs subsets. (D) Bar plot displaying upregulated GO terms in the FB0 compared to other subsets. Bars represent *z*‐score values, and dots represent −log10(adjusted *p*‐value). (E) Bar plot displaying upregulated GO terms in the FB1 compared to FB0 subsets. Bars represent *z*‐score values, and dots represent −log10(adjusted *p*‐value). (F) Scatter plot showing the upregulated genes (red) and downregulated genes (blue) in the FB1 compared to FB0 subsets. (G) Upregulated KEGG pathway in FB1 compared to FB0. Bars represent *z*‐score values, and dots represent −log10(adjusted *p*‐value).

Fibroblasts play a crucial role in vascular wall maintenance and remodeling [36]. UMAP visualization revealed that fibroblasts were primarily distributed in the PA region, with fewer present in the plaque core (Figure 5A,B). Based on enriched genes, fibroblasts were classified into three subpopulations: Cluster 0 highly expressed fibrosis‐related genes (*MFAP5*, *FBN1*, *IGFBP5*); Cluster 1 was enriched in antigen presentation genes; and Cluster 2 predominantly expressed ribosomal genes (Figure 5C). The Cluster 0 upregulated ECM and collagen‐related functions, suggesting a basal fibroblast state, with additional activation in transmembrane receptor protein kinase and insulin‐like growth factor receptor signaling pathways (Figure 5D). The expression of *ACTA2* in Cluster 2 fibroblasts suggests their potential myofibroblast phenotype (Figure S5A). Myofibroblasts promote disease by forming the plaque's fibrous cap and contributing to the lipid core, but they can also destabilize the plaque by remodeling the ECM and promoting inflammation and calcification [37]. Compared to Cluster 0, Cluster 1 exhibited enhanced leukocyte activation, proliferation, and MHC complex functions (Figure 5E). Volcano plots highlighted upregulated antigen presentation genes (*HLA‐DRB1*, *HLA‐DRB5*, *CD74*, *HLA‐DRA*), inflammatory markers (*JUNB*, *ZFP36*, *NR4A1*, *ATF3*, *IER2*), and chemokines (*CXCL14*, *CXCL2*), alongside downregulated fibrotic genes (*FBN1*), implicating Cluster 1 in immune‐inflammatory responses (Figure 5F). KEGG analysis further linked Cluster 1 to Th17/Th1/Th2 differentiation, TNF signaling, antigen presentation and IL‐7 pathways, suggesting its role in promoting plaque instability in the PA region (Figure 5G).

### 2.5 Macrophages in AC Region Exhibit Enhanced Phagocytic Function, While T‐Cell Subsets Show Minimal Regional Distribution Differences

**FIGURE 6:**
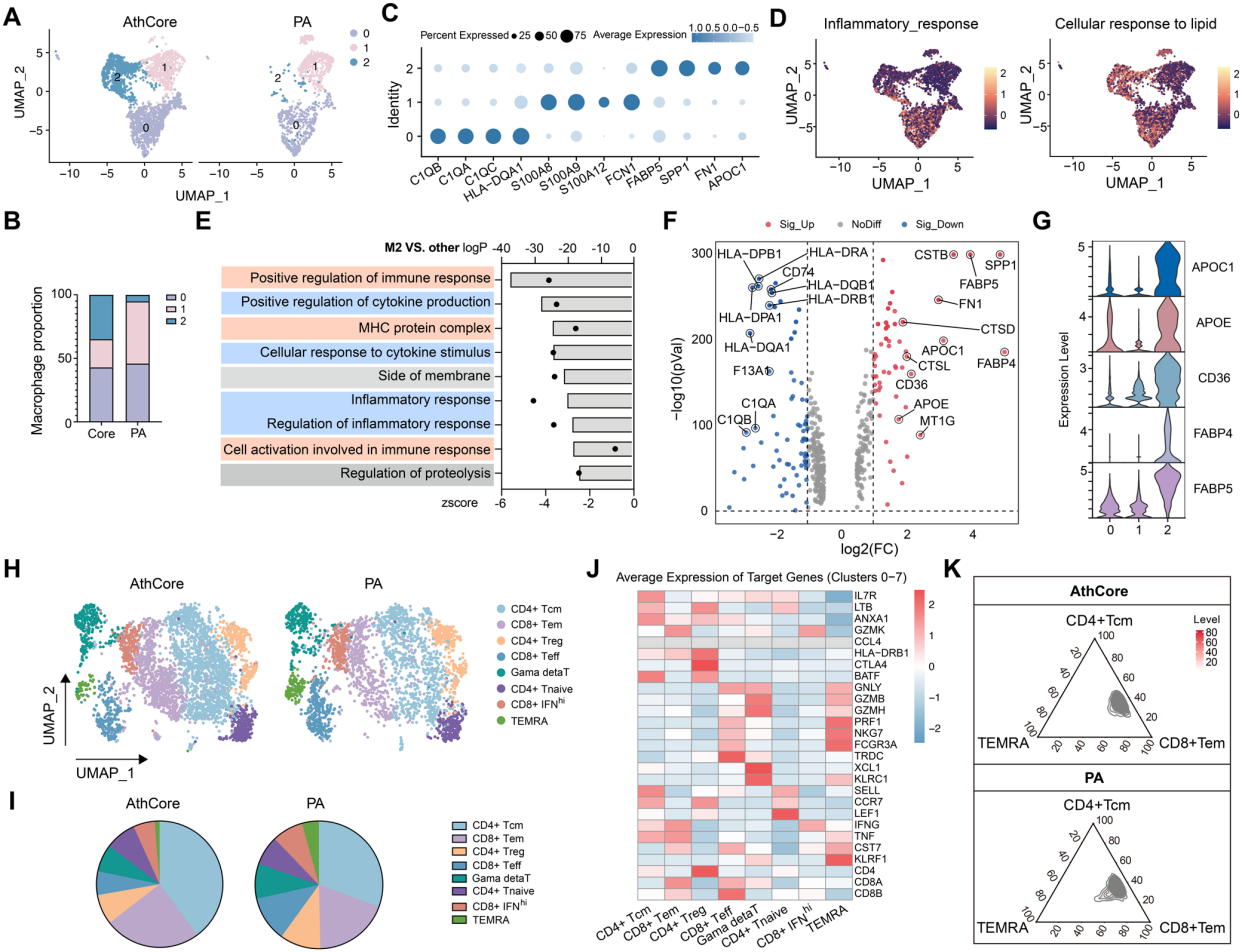
Macrophages in the AC region exhibit enhanced phagocytic function, while T‐cell subsets show minimal regional distribution differences. (A) UMAP plot of macrophages in the AC and PA regions. (B) Proportions of the different M ø subsets. (C) Dot plot displaying marker genes in the indicated groups. (D) Scaled module score based on functional gene sets. (E) Bar plot displaying downregulated GO terms in the M ø 2 compared to other subsets. Bars represent *z*‐score values, and dots represent −log10(adjusted *p*‐value). (F) Scatter plot showing the upregulated genes (red) and downregulated genes (blue) in the M ø2 compared to other subsets. (G) Violin plot illustrating that gene expression related to lipid metabolism in the indicated M ø subsets. (H) UMAP plot of T cells in the AC and PA regions. (I) Proportions of the different T cell subsets. (J) Heatmap showing the marker gene expression for each T cell subset. (K) Ternary density plots comparing the distribution of T cells between the AC and PA regions.

Macrophages were categorized into three subpopulations based on enriched genes (Figure 6A,C). UMAP visualization revealed distinct distributions, with Cluster 2 predominantly localized in the AC region (Figure 6A,B). Module scoring demonstrated that Cluster 0 scored highly in inflammatory responses, while Cluster 2 and parts of Cluster 0 cells showed elevated lipid metabolism activity (Figure 6D). GO analysis indicated that Cluster 2 downregulated immune responses, cytokine production, and MHC complex functions compared to the other clusters (Figure 6E). Volcano plots confirmed reduced expression of antigen presentation and complement genes in Cluster 2, alongside upregulated pro‐inflammatory markers (*SPP1*), ECM degradation regulators (*CTSL*, *CTSD*, *CSTB*), and lipid metabolism genes (*FABP5*, *FABP4*, *APOC1*, *CD36*, *APOE*), highlighting its central role in lipid processing within the AC region (Figure 6F,G). The above analysis indicated that Cluster 2 is foamy cells. Foam cells are the hallmark of the initiation of atherosclerosis [38]. Excessive accumulation of foam cells in the AC region leads to necrosis within atherosclerotic plaques.

T cells were subdivided into eight subsets: CD4+ Tcm, CD8+ Tem, CD4+ Treg, CD8+ Teff, gamma delta T, CD4+ naive, CD8+ IFN, and TEMRA cells (Figure 6H,J). UMAP revealed minor distribution differences, with TEMRA T cells slightly more abundant in the PA region, potentially driving plaque progression via pro‐inflammatory mechanisms (Figure 6H,I,K). In summary, the distribution and functional differences of various T cell subsets across different plaque regions are minimal.

## 3 Discussion

Our study systematically analyzed the spatial and functional heterogeneity of various cell types in human carotid plaques using single‐cell RNA sequencing data, exploring their roles in plaque. The findings revealed distinct cellular distributions between PA and AC regions. A subset of ECs contributing to pathological neovascularization was prominent in the AC region, while the PA region exhibited a reduction in contractile VSMCs and an increase in osteogenic VSMCs, collectively contributing to vascular remodeling.

Atherosclerosis is a complex vascular pathological process involving dynamic changes and interactions among multiple cell types. AC region is a hypoxic microenvironment. Consistent with previous studies, the AC is composed of lipid‐laden macrophages, making it mechanically prone to rupture [39, 40]. Our study revealed that macrophage cluster 2 in the plaque core region exhibits typical foam cell characteristics: upregulation of lipid metabolism‐related genes and downregulation of immune response and antigen presentation functions. These macrophages transform into foam cells by engulfing lipoproteins and subsequently release inflammatory mediators and cytokines. Moreover, hypoxia induces the formation of immature, fragile, pathological neovessels that are relatively active in the AC. EC4 was significantly enriched and displayed a pro‐fibrotic/pro‐calcific phenotype. This subpopulation promotes ECM remodeling by upregulating ECM‐related genes, thereby contributing to pathological neovascularization. When hemodynamic changes occur in the blood flow both inside and outside the vessel lumen, the neovessels within the plaque are often affected, leading to functional impairment. Consequently, red blood cells and inflammatory cells infiltrate the plaque, triggering intraplaque hemorrhage and the aggregation of inflammatory cells. This may reduce plaque stability and ultimately lead to adverse cardiovascular events [41]. Current studies have found that intraplaque neovascularization is an important predictor of plaque hemorrhage and rupture [42]. Additionally, our study found that VSMC4 was highly enriched in the AC region and maintained a typical contractile phenotype. During plaque progression, inflammatory stimuli drive the migration of contractile VSMCs from the media to the intima, where they undergo phenotypic switching into synthetic VSMCs. These transformed VSMCs not only exhibit enhanced proliferation but also secrete abundant ECM proteins, playing a critical role in the initiation and progression of atherosclerosis [43, 44].

In the PA region, various cell subtypes are primarily involved in vascular remodeling. EC‐dependent inflammatory regulation is a key driver of atherosclerosis, while both innate and adaptive immune responses are implicated in plaque progression [45]. The PA region located approximately 1 cm proximal to the AC tissue is in a relatively hypoxic state. The PA region was dominated by EC1, EC2, EC5, and EC6, all highly expressing immune‐regulatory genes and partially expressing cytokine‐related genes, indicating their active role in inflammatory responses. Notably, EC5 exhibited high expression of *CXCL2*, a pro‐inflammatory chemokine that may exacerbate plaque inflammation by recruiting leukocytes. These functional changes amplify the inflammatory cascade and are closely associated with the progression of atherosclerosis. In the PA region, the VSMC1 subset predominantly displayed an osteogenic phenotype, with markedly reduced contractility and increased expression of bone development‐related and ECM genes, driving vascular ectopic calcification. Osteogenic VSMCs promote calcification, exacerbating atherosclerosis and plaque instability [46]. Previous studies have shown that the expression of the *DLX5* gene can regulate the process of osteogenic differentiation in certain diseases [47, 48], which is highly expressed in the VSMC1 subpopulation. Moreover, VSMC1 also expressed genes that inhibit calcification, reflecting a complex regulatory network in plaque calcification. Adventitial fibroblasts are metabolically active cells that play a significant role in the development of atherosclerosis [49]. In this study, fibroblasts were primarily distributed in the PA region and could be classified into three functionally distinct subpopulations. Cluster 0 exhibited high expression of fibrosis‐related genes and upregulation of ECM and insulin‐like growth factor receptor signaling pathways, likely representing a basic state of fibroblasts. Studies suggest that resident adventitial fibroblasts may be among the first cells in the vascular wall to respond to inflammatory and environmental stimuli [50]. In our study, Cluster 1 showed significant upregulation of antigen presentation, leukocyte activation, and inflammation‐related genes, with enrichment in Th17 and TNF signaling pathways, indicating its critical role in the inflammatory response within atherosclerotic plaques. These findings reveal the dual role of fibroblasts in atherosclerosis: participating in fibrotic processes to maintain vascular wall structure while also influencing plaque progression through inflammatory regulation.

Our study has certain limitations, primarily that key conclusions are largely based on single‐cell transcriptomic sequencing data analysis. Specifically, by bioinformatically mining the gene expression profiles of different EC subpopulations, we inferred their distribution differences between the PA and AC regions, functional tendencies, and associations with plaque inflammation or vascular remodeling. However, these findings at the transcriptional level (mRNA expression) have not been directly validated through independent experimental methods. For example, proteomic assays have not been used to confirm the actual protein expression levels of key molecules in corresponding cell subpopulations. Functional experiments to elucidate the causal roles of specific subpopulations or molecules in plaque inflammation regulation, protective responses, or pathological remodeling have also not been conducted. In addition, due to the relatively low sequencing depth of this dataset, fewer genes were detected. Therefore, we chose a lower threshold when filtering genes in order to retain more biologically meaningful ones. It may lead to false positive results. Future data from deeper sequencing may provide more compelling research conclusions.

To address these limitations, follow‐up studies require more in‐depth validation and functional verification across multiple dimensions. Multi‐omics integration (e.g., matched single‐cell proteomic sequencing or spatial proteomics) should be employed to validate the consistency of protein expression identified in transcriptomic analysis and determine the actual translation levels of key molecules in target cell subpopulations. Only through such experimental validation and functional analysis can associative inferences based on transcriptomic data be transformed into causal conclusions, ultimately providing more reliable scientific evidence for precision interventions in atherosclerotic plaques.

## 4 Conclusion

The pathological neovascularization mediated by EC subpopulations in the AC region, along with the reduced contractile VSMCs and increased osteogenic VSMCs in the PA region, are factors that promote plaque instability. These identified subsets of cellular population provide novel molecular and cellular insights into the mechanisms of pathological vascular remodeling and potential targets for therapeutic intervention.

## 5 Methods

### 5.1 Selection of Patients

Tissue specimens were harvested from the atherosclerotic core—defined as the region with the greatest plaque burden, typically adjacent to the carotid bifurcation—extending nearly to full thickness while preserving the adventitia, as determined by the surgeon. Subsequently, during the dissection phase of the endarterectomy, a proximal arterial segment located approximately 1 cm from the AC was excised from the same patient. This protocol yielded three biological replicates each of the AC and the PA region, derived from three patients presenting with asymptomatic Type VII calcified plaques (Table S1).

### 5.2 RNA Sequence Data Processing

The scRNA‐seq data of GSE159677 was retrieved from the Gene Expression Omnibus (GEO) database. GSE159677 was performed on GPL18573, Illumina Next‐Seq 500 ( *Homo sapiens* ), containing three human calcified AC plaques and the matched PA portions of carotid artery. Count matrices were processed and integrated using Seurat (version 4.4.0) and Harmony (version 0.1.1) R packages. Cells were included with detected genes ranging from 350 to 5500 and a mitochondrial gene ratio of less than 5%. 10,000 cells were used for analysis in both the PA and the AC region. Data normalization and variance stabilization were carried out with the SCTransform function. After principal component analysis (PCA), the “FindClusters” function was used to classify the cells into different clusters followed by nonlinear dimensional reduction methods used for visualization, including uniform manifold approximation and projection (UMAP) and t‐distributed stochastic neighbor embedding (tSNE). The resolution for total cells is 0.1, ECs is 0.15, VSMCs is 0.4, macrophages is 0.1, and T cells is 0.3.

### 5.3 Differential Expression Analysis and Enrichment Analysis

Differentially expressed genes (DEG) were identified between clusters using the FindAllMarkers and FindMarkers functions. The Wilcoxon rank‐sum test was applied to compute *p* values, and Bonferroni correction was used to adjust *p* values for all genes. The adjusted *p* < 0.05 and |log2foldchange| > 0.25 was set as the threshold for significantly differential expression. Gene Ontology (GO) enrichment analyses were performed on Metascape (http://metascape.org). A list of DEGs was uploaded and statistically enriched GO terms were returned. *Z*‐score was calculated with GOplot package in R to predict the activation state for each GO term. A term was considered to be significantly changed with *Z*‐score > 2 or < −2 and adjusted *p* < 0.01.

### 5.4 Calculation of Functional Gene Set Module Score

AddModuleScore function in Seurat was used to calculate the average expression levels of each specific gene set on a single cell level, subtracted by the aggregated expression of control gene sets. All analyzed genes are binned based on averaged expression, and the control genes are randomly selected from each bin. Functional gene sets were related to leukocyte chemotaxis, MHC protein complex, tissue regeneration, sprouting angiogenesis, extracellular matrix assembly, regulation of tissue remodeling, and cellular response to lipid.

### 5.5 Pseudo‐Time Trajectory Analysis

To investigate the differentiation trajectory of EC and VSMC subsets, we utilized the R package Monocle (version 2.26.0) and Monocle3 (version 1.3.1). The cellular trajectory analysis was performed using the learn_graph function. Briefly, after inputting the Seurat object with clustering information, high‐variable genes were calculated, and those with a *p* < 0.01 and log2 (fold change) > 0.25 were selected as ordering genes. The dimensional reduction was done with the DDRTree algorithm. The cellular trajectory was generated using the order_cells function and the plot_cell_trajectory function. The results of Pseudo‐time analysis were visualized on the UMAP dimensional scatter plot. The pseudo‐temporal order of cells was constructed after choosing the starting point of the cellular trajectory. The one‐dimensional pseudo‐temporal distribution of cells was plotted with density function.

### 5.6 Tissue Specimens

The human atherosclerotic plaque tissue samples obtained during the surgery were rapidly frozen in liquid nitrogen‐cooled isopentane and stored at −70°C in a refrigerator until they were used for immunofluorescence staining. The study was approved by the Institutional Ethics Committee of the Second Affiliated Hospital, Zhejiang University School of Medicine (approval no. [2025] ethics review no. 1550).

### 5.7 Immunofluorescence Staining

The plaque samples were sectioned into 7 μm‐thick slices. The sections were washed three times with PBS, 10 min per wash. Subsequently, sections were blocked and permeabilized for 1 h in PBS containing 10% goat serum, 3% BSA, 1% Triton X‐100, and 0.2% Tween 20. Next, the sections were incubated overnight at 4°C with primary antibodies: goat anti‐CD31 (1:200, AF3628, R&D Systems), mouse anti‐CXCL12 (1:100, MAB310, Novus Biologicals), and rabbit anti‐COL8A1 (1:100, 17,251–1‐AP). After that, the plaque sections were washed three times with PBS, 5 min per wash. Then, the sections were incubated with cross‐adsorbed fluorescent secondary antibodies (1:500, Alexa Fluor, Invitrogen) at room temperature in the dark for 1 h, followed by mounting with DAPI‐containing mounting medium (ab104139, Abcam). Finally, the samples were observed using a confocal microscope (Leica DMi8).

## Funding

This work was supported by the National Natural Science Foundation of China (82001302), Natural Science Foundation of Zhejiang Province (LQ21H090012), Zhejiang Provincial Administration of Traditional Chinese Medicine Science and Technology Plan Project (GZY‐ZJ‐KJ‐23077).

## Ethics Statement

This study was approved by the Institutional Ethics Committee of the Second Affiliated Hospital, Zhejiang University School of Medicine (approval no. [2025] ethics review no. 1550).

## Conflicts of Interest

The authors declare no conflicts of interest.

## Supporting information


**Figure S1:** (A–C) Scaled module score of leukocyte chemotaxis, extracellular matrix assembly and sprouting angiogenesis.

**Figure S2:** (A) Violin plot of marker gene expression for the seven EC clusters. (B) Violin plot illustrating that genes expression related to quiescent endothelial cells in different subsets.

**Figure S3:** (A) Heatmap showing gene expression differences in the indicated EC subsets at different pseudotime stages. (B) Violin plot illustrating that genes expressed at different pseudotime in the AC and PA plaques. (C–E) Ridge plot of different gene expression in the indicated EC subsets. (F) Immunofluorescence staining of CD31/COL8A1 (upper) and CD31/CXCL12 (lower) in human carotid plaque. Scale bar: 50 μm.

**Figure S4:** (A) Bar plot displaying upregulated and downregulated GO terms in the VSMC4 compared to other subsets. Bars represent z‐score values, and dots represent −log10(adjusted *p*‐value). (B) Violin plot illustrating *KRT4* expression in VSMC subsets. (C) Violin plot illustrating that genes expression related to calcification in the indicated VSMC subsets.

**Figure S5:** (A) Violin plot illustrating that *ACTA2* expressed in different FBs subsets.

**Table S1:** Patient basic information and plaque calcification information.

## Data Availability

The data that support the findings of this study are openly available in GEO database at GSE159677.
