# Supplementary figures and images for: Inflammatory Neovascularization and Vascular Remodeling Associated With Carotid Plaque Destabilization
Source: CNS Neurosci Ther. 2026 Jun 19;32(6):e70992. doi: 10.1002/cns.70992 (PMC13281155; doi:10.1002/cns.70992)

**A**

Leukocyte chemotaxis

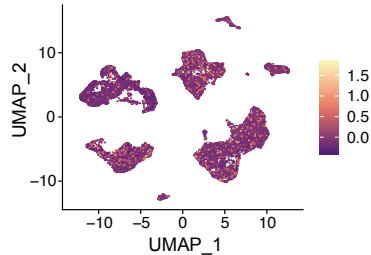**B**

Extracellular matrix assembly

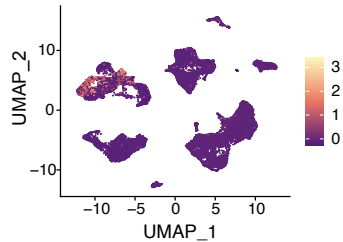**C**

Sprouting angiogenesis

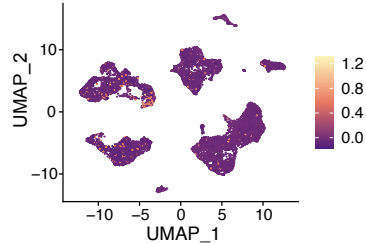

Supplement: Supplementary file 1 — Figure S1: (A–C) Scaled module score of leukocyte chemotaxis, extracellular matrix assembly and sprouting angiogenesis. [file CNS-32-e70992-s004.pdf]

**A**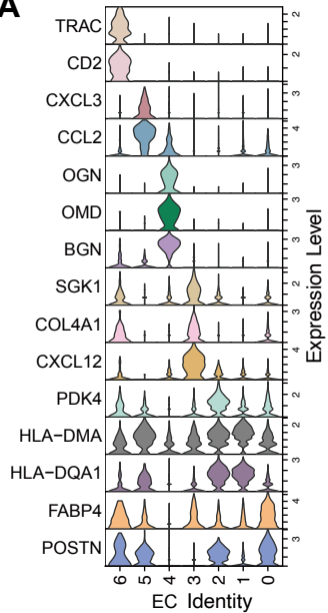**B**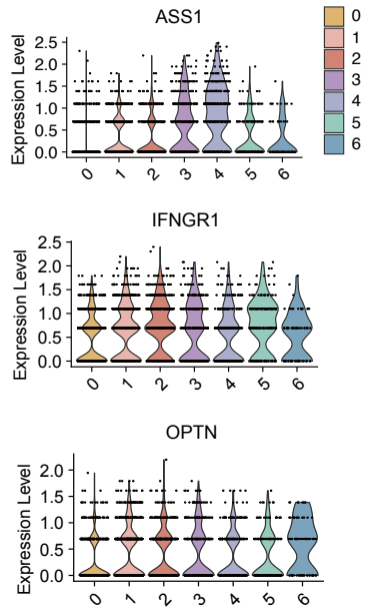

Supplement: Supplementary file 2 — Figure S2: (A) Violin plot of marker gene expression for the seven EC clusters. (B) Violin plot illustrating that genes expression related to quiescent endothelial cells in different subsets. [file CNS-32-e70992-s003.pdf]

**A**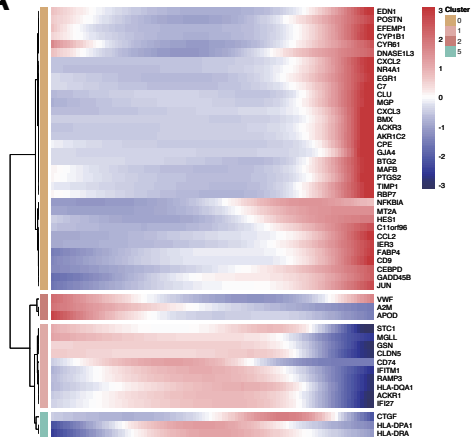**B**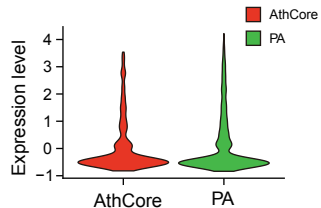**C**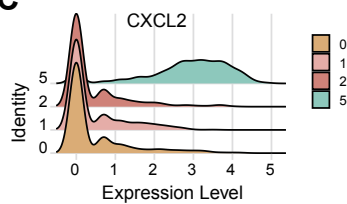**D**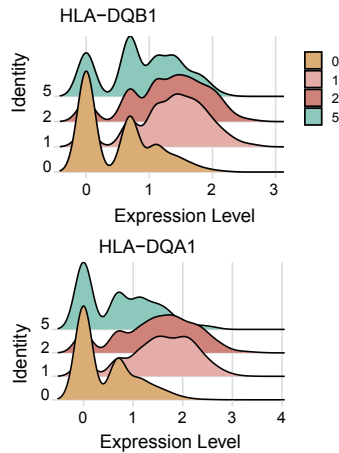**E**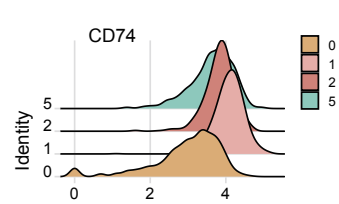**F**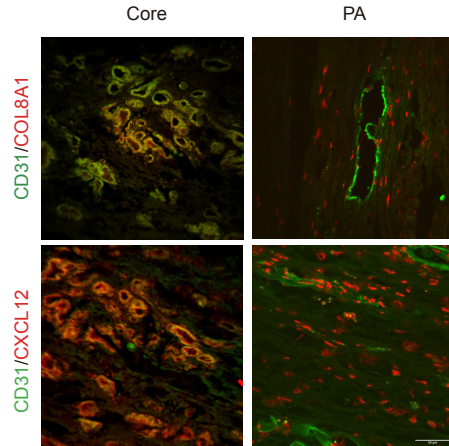

Supplement: Supplementary file 3 — Figure S3: (A) Heatmap showing gene expression differences in the indicated EC subsets at different pseudotime stages. (B) Violin plot illustrating that genes expressed at different pseudotime in the AC and PA plaques. (C–E) Ridge plot of different gene expression in the indicated EC subsets. (F) Immunofluorescence staining of CD31/COL8A1 (upper) and CD31/CXCL12 (lower) in human carotid plaque. Scale bar: 50 μm. [file CNS-32-e70992-s006.pdf]

**A**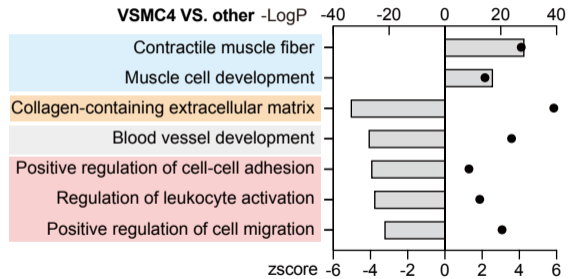**B**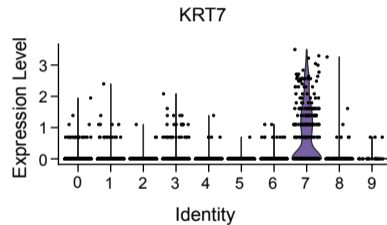**C**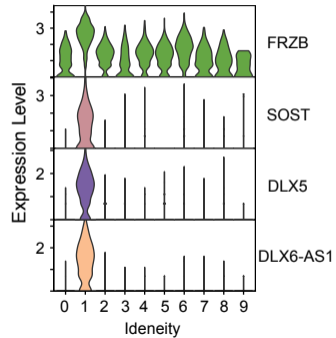

Supplement: Supplementary file 4 — Figure S4: (A) Bar plot displaying upregulated and downregulated GO terms in the VSMC4 compared to other subsets. Bars represent z‐score values, and dots represent −log10(adjusted p‐value). (B) Violin plot illustrating KRT4 expression in VSMC subsets. (C) Violin plot illustrating that genes expression related to calcification in the indicated VSMC subsets. [file CNS-32-e70992-s005.pdf]

**A**

ACTA2

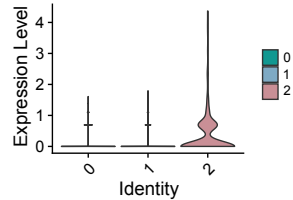

Supplement: Supplementary file 5 — Figure S5: (A) Violin plot illustrating that ACTA2 expressed in different FBs subsets. [file CNS-32-e70992-s001.pdf]
