# Supplementary material for: Inflammatory Neovascularization and Vascular Remodeling Associated With Carotid Plaque Destabilization
Source: CNS Neurosci Ther. 2026 Jun 19;32(6):e70992. doi: 10.1002/cns.70992 (PMC13281155; doi:10.1002/cns.70992)
Supplement: Supplementary file 6 — Table S1: Patient basic information and plaque calcification information. [file CNS-32-e70992-s002.docx]

Table S1: Patient basic information and plaque calcification information

| Patient | Age | Smoker | Diabetes | Hypertension | Dyslipidemia | Stain | Symptomatic | AHA Classification |
| --- | --- | --- | --- | --- | --- | --- | --- | --- |
| 1 | 82 | Yes | Yes | Yes | Yes | Yes | No | Type VII Calcified |
| 2 | 87 | No | Yes | Yes | Yes | Yes | No | Type VII Calcified |
| 3 | 65 | Yes | No | Yes | Yes | Yes | No | Type VII Calcified |
